# Brief assessments and screening for geriatric conditions in older primary care patients: a pragmatic approach

**DOI:** 10.1186/s40985-018-0086-7

**Published:** 2018-05-01

**Authors:** Laurence Seematter-Bagnoud, Christophe Büla

**Affiliations:** 1 Service of Geriatric Medicine and Geriatric Rehabilitation, University of Lausanne Hospital Center, Mont Paisible 16, CH-1011 Lausanne, Switzerland; 2 Institute of Social and Preventive Medicine, University of Lausanne, Lausanne, Switzerland

**Keywords:** (3–10) Screening, Older adults, Geriatric assessment, Primary care

## Abstract

This paper discusses the rationale behind performing a brief geriatric assessment as a first step in the management of older patients in primary care practice. While geriatric conditions are considered by older patients and health professionals as particularly relevant for health and well-being, they remain too often overlooked due to many patient- and physician-related factors. These include time constraints and lack of specific training to undertake comprehensive geriatric assessment. This article discusses the epidemiologic rationale for screening functional, cognitive, affective, hearing and visual impairments, and nutritional status as well as fall risk and social status. It proposes using brief screening tests in primary care practice to identify patients who may need further comprehensive geriatric assessment or specific interventions.

## Background

This paper describes the epidemiological rationale and scientific background for performing a systematic assessment of older patients consulting a family physician and proposes a pragmatic approach to screen for several frequent and usually under-detected geriatric conditions.

## Why would a systematic assessment of geriatric conditions be useful in caring for older patients?

Given the aging of the population, primary care practitioners will manage an increasing number of older patients who often have multiple health problems. Besides the complexity of managing multiple morbidities, other concurrent factors can make care more difficult in older patients. First, health problems are less obvious to diagnose because of an atypical presentation or because of communication problems due to hearing loss or cognitive impairment. Banalization of symptoms considered by patients or health professionals as features of normal aging also frequently prevents health problems such as incontinence or cognitive impairment from being identified [1]. Indeed, the quality of ambulatory care in primary care patients with geriatric conditions has been shown to be lower than in those with non-geriatric conditions [2]. Finally, problems outside the traditional medical domain, such as those related to the psycho-social status or the environment, increase in importance in older patients because they frequently coexist with health problems and interfere with their management. Yet, physicians often lack guidance to assess these impairments [3, 4].

Comprehensive geriatric assessment (CGA) was developed as a multidimensional and structured approach aimed at the identification and management of these problems in older patients. The CGA process is not only limited to the evaluation of an older patient’s global health status, with the mere mention of the presence of medical and functional problems, but also includes the identification of patient’s resources, capacities, and preferences [4]. CGA is intended to help the physician select and prioritize therapeutic interventions that are best suited for a given patient. These latter aspects of CGAs are not addressed in this article, which proposes brief tools to screen for a selected set of geriatric conditions. In contrast to hospital or long-term care settings, where an interdisciplinary team usually performs a CGA, it is typically undertaken by geriatricians and/or trained gerontological nurses in the primary care setting [5, 6]. Such a comprehensive assessment of physical and mental health and social situation usually takes more than 60 min to complete, with additional time needed to define interventions [7].

The diagnostic yield of a CGA in identifying geriatric conditions and improving the management of unmet patient needs has been demonstrated in several populations, including the community and primary care practice [2, 8, 9]. In particular, a study in general practice highlighted that such an approach is particularly worthy when the patient-physician relationship has persisted less than 2 years, with a twofold number of problems newly found independent of the quality of the relationship [3]. Nonetheless, evidence to support the use of CGA in primary care practice remains weak because it has been less well studied.

## What are the target group and objectives of a brief geriatric assessment?

Depending on the global health status of the patient, the objectives may range from health promotion to screening or early detection and to subsequent decision-making prior to therapeutic interventions. As conceptualized in the new public-health framework for healthy aging defined by the World Health Organization (WHO), target groups are defined based on the older person’s intrinsic capacity (mental and physical) and functional ability. Physical and mental capacity as well as functional ability corresponds to health-related attributes that enable people to do what they value. Fit older persons, who have fewer chronic diseases and a high intrinsic capacity and functional ability, are an appropriate target for health promotion and preventative interventions, but universal CGA in this large group would be too time-consuming. By contrast, it has been shown that older persons who are already disabled need integrated care and do not benefit from a CGA performed during preventive home visits [10]. A CGA is likely more suited to the group in between, i.e., older people with two or more chronic conditions but no or minimal disability, with the aim of slowing the decline in capacity [11].

Patients who will undergo surgical or oncological interventions represent another potential target for a brief CGA to identify those at risk for adverse outcomes and to propose follow-up through the peri-operative period, with a reduction in complication rates and length of stay [12, 13].

Some consensus has emerged to propose a two-step approach for a brief CGA targeting primary care patients aged 75 to 80 years and older with two or more comorbid conditions, or before surgical or oncological interventions [8]. For instance, the EASY-Care tool helps primary care practitioners identify frail older patients who then have a comprehensive assessment by a specialized nurse during a home visit [14]. Similarly, the British Geriatrics Society recently proposed an approach using the PRISMA questionnaire, combined with an evaluation of gait and mobility [15].

In this paper, we propose a pragmatic approach for primary care visits and focus on the early detection of several geriatric conditions rather than on issues related to health promotion in older adults. Additionally, this paper does not extend to the identification of patients’ resources and preferences, which are part of CGA and are needed to decide how to best address identified problems with appropriate and individualized interventions [16].

## What might be the benefits for the patient?

Interest in managing specific chronic health problems has led to the development and testing of disease-specific interventions, an approach that is irrelevant in most older persons who have multiple health problems [17]. Conversely, CGA proposes an approach that focuses on function-related outcomes and addresses the problem of the poor correlation between symptoms and underlying causes in older persons with multiple chronic diseases. Moreover, CGA better addresses the increasing variability of individual expectations toward care as people age.

When initiated in community-dwelling, hospitalized, older persons, CGA reduced disability, extended home stay, and reduced the institutionalization rate by 20% over the following 12 months compared to usual care [18]. Among older persons receiving formal home care, CGA led to a decreased risk of hospital and nursing home admission. As health care costs were examined, the higher supply of home care interventions was more than balanced by the reduction in institutional costs in hospital and nursing homes [19].

In general practice, a study indicated that about half of problems newly identified through CGA were successfully managed by the physician at 12 months [3]. Most studies reported improvement in either the quality of care (e.g., indicators of fall-risk assessment and management [20]) or in patients’ quality of life, while a few demonstrated a decrease in hospital admission rates [21].

Regarding CGA’s cost-effectiveness, a meta-analysis of previous studies demonstrated conflicting results depending on the target population and specific interventions [18]. Evidence is still lacking regarding the economic benefits of a brief geriatric assessment performed in primary care [22].

## Which dimensions should be assessed?

A systematic review of factors associated with the occurrence of disability identified functional, cognitive, affective, and social problems as well as, to a lesser extent, lifestyle habits and sensory impairments as modifiable risk factors for functional decline [23]. The domains usually selected for inclusion in CGA are risk factors that meet the usual criteria for appropriate screening. Impairment is frequently overlooked, the screening test is sensitive and specific enough, the diagnostic procedures have an acceptable risk to benefit ratio, and the condition may be improved by interventions. A study that specifically investigated common unmet needs identified by older people themselves proposed five priority domains: (a) hearing and vision; (b) physical ability, including functioning in activities of daily living (ADL), mobility, and falls; (c) incontinence; (d) cognition; and (e) emotional situation [8]. Assessing nutrition and the patient’s social situation is frequently added to this list [24]. These dimensions have also been identified by a panel of experts who contributed to the WHO guidelines on integrated care for older people [4]. The guideline recommends assessing decline in physical and mental capacities (mobility, nutrition, vision, hearing, cognition, and depression) as well as assessing two geriatric syndromes (urinary incontinence and risk for falls).

## How could CGA fit into primary care consultations?

Time investment is a major limitation in applying a full CGA in a busy practice. In order to fit into primary care physicians’ tight schedule, several combinations of short instruments to identify geriatric conditions have been proposed [8, 24]. These instruments allow basic multidimensional screening in about 15 min and target patients who might benefit from a further in-depth assessment by the primary care physician or referral to a geriatrician for further assessment and management.

The psychometric properties of these instruments as well as their impact on clinically relevant outcomes have not been thoroughly examined. Consequently, there is little evidence to promote the use of one over another or a combination of their components. The choice should rely on issues of practicability and on the estimated suitability to the profile of the targeted population and particular health system.

In parallel, several instruments have been developed to help identify frailty in primary care [7, 8, 14, 15, 24], which include domains similar to those included in the brief CGA, consistent with the fact that frailty and functional decline are distinct but overlapping concepts that share common risk factors [25, 26].

This article proposes a pragmatic approach for primary care physicians to assess a selected set of dimensions when caring for older patients. Although this selection is not evidence-based, all proposed dimensions share common features in terms of prevalence of impairment, frequent under-detection, existing valid brief screening tests, and corresponding effective therapeutic or supportive interventions. For several of these dimensions, referral to the summary of evidence issued by the US Preventive Services Task Force (USPSTF) [27] and/or the Canadian Task Force on Preventive Health Care [27, 28] has been used. In several cases, the task forces considered the evidence from the literature regarding the impact of screening and subsequent management on clinical outcomes as being too scarce to support a formal recommendation. However, the very same dimensions have been included in the WHO guidelines based on expert consensus regarding the balance of benefit and harm, the match with older persons’ values and preferences, and the cost and feasibility of assessment [4]. Such a brief evaluation might be completed in about 15 min. Although the precise timeline for repeating the evaluation is not defined, a yearly assessment has most frequently been proposed [29].

## Screening for functional impairment

A core feature of CGA is the evaluation of a patient’s functional ability, i.e., how he or she is able to perform usual ADL. Assessment of daily function is decisive to identify functional decline because it reflects the consequences of health problems. Moreover, functional status provides essential information about prognosis and the future functional trajectory [30]. Finally, the early identification of functional difficulties and search for etiology are the first steps toward interventions to prevent further loss of function, restore function, and address resulting needs for support or personal care.

Functional status is assessed as a difficulty or impairment in basic and instrumental ADLs, as detailed in Table 1. Instrumental ADLs are more complex activities that require higher neuropsychological capacity than physical self-maintenance. They are therefore usually affected before basic ADLs [31].

**Table 1:** Description of basic and instrumental activities of daily living

Basic activities of daily living include the following [73]:	Instrumental activities of daily living include the following [31]:
Bathing	Use the telephone
Dressing	Use public transportation
Toileting	Do grocery shopping
Transferring (in-out of bed/chair)	Prepare meals
Continence (bladder, bowel)	Handle own medication
Eating	Handle finances
	Do housekeeping
	Do laundry

Functional difficulties in instrumental ADLs are strongly related to cognitive function. For instance, the onset of impairment in four instrumental ADLs (using the phone, using public transportation, using own medications, and handling finances) has been shown to be associated with a 4 (when one instrumental ADL is impaired) to 10 (with 3 or 4 impaired ADLs) times higher odds of being diagnosed with dementia in the following 12 months [32]. Because a full assessment of basic and instrumental ADLs can be difficult during a clinical encounter, a first general question regarding the onset of difficulties in performing those tasks might be useful [24]. However, there is often a discrepancy between self-reported and actual performance in ADLs. Because of this, asking relatives about any difficulties in ADLS might be useful.

## Screening for cognitive impairment

### Epidemiology

The prevalence of dementia increases steeply with age, ranging from less than 5% in adults aged 65 to 70 years to up to 30–40% in those aged 90 years and older [33]. Despite a decrease in age-specific incidence rates of dementia, the rise in longevity implies a substantially growing number of persons with dementia over the next decades [34]. Cognitive impairment threatens functional independence and imposes a major burden on the older person, their caregivers, and the health care system.

### Under-diagnosis

Diagnosing dementia in the primary care setting might be challenging. In addition to the general difficulties mentioned in the introduction, a specific one is that early symptoms of dementia may not be apparent and are sometimes even concealed during short office visits initiated for other complaints. As a consequence, a substantial proportion of subjects with dementia remain undiagnosed until later stages [35, 36].

### Rationale for screening

Given the absence of effective pharmacological treatments for Alzheimer-related disease and the fear of the potential negative and stigmatizing effects of such diagnoses, current evidence is considered insufficient to encourage systematic screening for dementia in older patients [27, 28]. However, this position does not recognize the many benefits of a proactive detection of cognitive impairment, which permits the diagnosis of reversible causes of memory problems (such as depression), an appropriate management of other comorbidities, and also allows patients and relatives to prepare for future care and decisions [36].

### Brief screening instrument

Among other tests, the Mini-Cog is appealing for the use in primary care practice because it can be completed in about 2–4 min with good sensitivity (73 to 99%) and specificity (75 to 93%) and does not depend on linguistic and educational background [35, 37]. It combines a three-item recall test with the clock drawing test. Impaired cognition is suspected whenever the patient is unable to recall any word or recalls one or two words with an abnormal clock drawing. Patients with positive Mini-Cog screening should be referred for more extensive neuropsychological testing.

## Screening for depression

### Epidemiology

Significant depressive disorders are found in about 10–15% of older persons [38]. The older population is particularly exposed to risk factors for depression such as health problems, sensory and cognitive impairment, adverse life events, bereavement, and social isolation.

### Under-diagnosis

Rates of detection and appropriate treatments remain low, with about half of depressed patients recognized as such by primary care physicians [38]. Compared to younger adults, older ones less often experience typical depressive symptoms, but are more likely to report physical problems, such as pain or insomnia, when depressed [39]. Impairment in decisional and memory capacity is not uncommon, underlying the complex bidirectional association between cognition and mood.

### Rationale for screening

The use of short screening questionnaires on anxiety and depression symptoms has been extensively tested in primary care. These questionnaires have been judged as acceptable by patients, most of whom agreed that primary care physicians should ask about mood and anxiety [40]. Regarding clinical benefits, primary care approaches that combine screening and intervention improve patients’ depressive symptoms, quality of life, and functional impairment [38, 41].

### Brief screening instrument

Very brief, 2-question tools are available that have excellent psychometric properties and seem especially convenient to use in primary care practice [42, 43]. The patient is asked whether, over the past 2 weeks, he/she has often had little interest or pleasure in doing things and whether he/she has often been bothered by feeling down, depressed, or hopeless. Negative answers to both questions essentially rule out depressive problems, whereas any positive answer should raise suspicion for depressive problems (sensitivity 95%, specificity 65% against clinical diagnostic interviews [42]) and trigger further assessment. Using more sophisticated coding of each answer (0 = not at all, 1 = several days, 2 = more than half the days, 3 = nearly every day) has resulted in improved specificity, without affecting the sensitivity too much (sensitivity 83% and specificity 90% for a score of 3 or more) [44].

## Screening for sensory impairments

### Epidemiology

Sensory impairments rank first on the list of geriatric impairments. About 30–45% of persons aged 75 years and older have hearing impairment that impacts their ability to communicate [17, 45]. Visual impairment also affects nearly 50% of adults over 75 years, and up to 10% of older persons report being unable to read newspapers, even with their glasses or lenses (USPTSF). The impact of these sensory impairments on functional trajectory is likely to be underestimated because it most usually occurs through reduced social contacts, psychological well-being, cognitive functioning, and a higher risk for falls as regards visual impairment [17, 46–48].

### Under-diagnosis

Visual and hearing impairments too often remain undiagnosed because they usually appear and progress insidiously in older persons and are still sometimes considered as normal consequences of aging. Proper management of visual and hearing conditions is also insufficient despite the availability of effective interventions. In particular, most older persons with significant hearing impairment do not have appropriate management or hearing aids [49]. Also, almost 4 out of 10 older persons have under-corrected refractive errors [50].

### Rationale for screening

Data about improved clinical outcomes following systematic screening of individuals without complaints are still too scarce [27]. Yet, a review of the evidence shows that screening and diagnostic procedures for sensory impairment are quite safe. Moreover, sensory function might be improved by surgical, corrective, or adaptive interventions, with a limited risk of complications and a positive impact on quality of life and well-being. Overall, these data suggest an overall benefit of screening, especially among older adults aged over 75 years. Similarly, studies about the management of hearing impairment suggest that clinical benefits are mostly seen in patients with moderate-to-severe impairment (> 40 db). Adults aged 75 years and older might therefore also be an appropriate target population to improve the efficiency of the screening process [27, 51].

### Brief screening instrument

#### Hearing impairment

The relevance of detecting mild (25 to 40 dB) hearing loss is uncertain regarding evidence of further clinical interventions; hence, a very first step in identifying patients with significant impairment might be to ask a single question about perceived hearing loss. This approach has a sensitivity and specificity of around 70% as compared to audiometry [17]. Among other tests, the whispered voice test seems best suited for use in primary care, with an overall good sensitivity (> 90%) and specificity (> 75%) as estimated in the USPSTF synthesis [27].

#### Visual impairment

Screening for visual impairment using the Snellen eye chart performs better than screening questionnaires [52]. An important caveat is that visual acuity testing identifies refractive errors well, but is inadequate for identifying early macular degeneration or early cataract.

## Screening for nutritional problems

### Epidemiology

Under-nutrition is not very frequent among community-dwelling adults aged 65 to 75 years, who are frequently overweight [53]. However, its prevalence increases sharply after 75 to 80 years and is likely triggered by factors such as chronic illnesses, medication, and socio-economic and psychological problems.

### Under-diagnosis

Involuntary weight loss, especially among overweight patients, frequently goes undetected. Yet, such weight loss predicts a poor functional trajectory and other adverse outcomes either through a direct pathway or because weight loss is a consequence of dental problems, or physical or mental illness (ref).

### Rationale for screening

The underlying assumption is that screening allows nutritional concerns to be identified early. Yet, the benefit of nutritional supplementation has not only been demonstrated in subjects considered “at risk” for malnutrition, but only in those with overt malnutrition [54].

### Brief screening instrument

Body mass index (BMI) is frequently used as a single screening measure, but it lacks specificity. For instance, a BMI less than 22 kg/m^2^ should already hint at possible malnutrition in an older person as it is associated with increased mortality [55]. A question about involuntary weight loss over the last months is a frequently used alternative, with a loss of 5% or more over a month or 10% or more over the last 6 months being used as cutoff to define malnutrition [56]. Brief instruments have also been proposed to identify patients who need further detailed nutritional assessments. These screening tools include the following items: appetite or appetite loss, weight loss, and BMI, and are sometimes combined with questions about acute disease. Their added value over monitoring patient’s weight and BMI is still unknown. Among these instruments, the Mini-Nutritional Assessment short-form (MNA-SF) has been developed specifically for older persons and is most frequently used [57].

## Fall-risk assessment

### Epidemiology

Overall, one in three persons aged 65 years and older falls every year, and one in 10 will have a significant injury, making falls a major threat to functional independence. Previous falls and activity restrictions resulting from subsequent fear of falling combine to further increase the likelihood of future falls by three times [58, 59].

### Under-diagnosis

In the absence of such injury, the occurrence of falling is seldom reported to the primary care physician. Yet, this simple information helps identify patients at risk for future falls [60].

### Rationale for screening

Detecting fall risk is a crucial component of a geriatric assessment because effective interventions to prevent future falls and limit their consequences are available. Patients with previous falls, but without gait or balance problems, should be counseled regarding secondary fall prevention (i.e., promotion of physical activity or consider a prescription of calcium and vitamin D), while those at higher risk should be fully assessed for risk factors, and individualized interventions should be proposed. A Cochrane review identified exercise programs, including Tai Chi, and home interventions as effective in reducing the rate of falls and the risk of falling by around 25 to 30% [61]. Community-based, multi-modal fall prevention programs are frequently available that simultaneously address risk factors identified in a patient.

### Brief screening instrument

Given that risk factors for falling encompass diseases, drugs, cognition, and sensory and gait impairment as well as environmental hazards, a comprehensive review is unsuitable as a first step, but the assessment of the patient’s global risk for falling should be part of a brief geriatric assessment.

Based on the American and British Geriatrics Societies guidelines and the input from health care providers, a fall prevention tool kit has been developed to support health care professionals in assessing and addressing fall risk [62]*.* A first set of questions identify whether the patient is at risk and investigates previous falls, fear of falling, and perception of unsteadiness when standing or walking. The performance in gait and balance is based on the up-and-go test (i.e., the patient gets up from the chair, walks 3 m, turns around, and sits back down) or by observing whether he or she stops walking when talking, a strong predictor of the probability of falling [63]. A simple measure of gait speed has also been shown to be a strong prognostic factor [64]. A gait speed less than 0.8 m/s (i.e., 5 s or more to walk 4 m at a usual pace) should trigger further assessment for future falls, frailty, and mobility impairment.

## Social isolation

### Epidemiology

Growing older often goes in parallel with a shrinking social network due to death of one’s peers, while reduced mobility might be an obstacle to social activities. Other societal factors, such as the move from intergenerational to single living, can increase the risk for social isolation in older persons [65]. Almost one in two older persons in Europe lives alone, and 10–20% report recurrent feelings of loneliness, most often when living alone and when reporting low social participation [66, 67].

### Rationale for screening

Social support is a major component to evaluate during CGA. This support is important because it buffers the effect of stressful events and improves the management of chronic diseases [65, 68, 69]. For instance, identifying potential social support in case of health problems has been associated with a lower likelihood of hospital use [70, 71]. However, older patients are not likely to discuss this issue spontaneously. For instance, in a study among primary care patients aged 65 years and older, only 15% of those reporting loneliness had mentioned it to their GP [66].

### Brief screening instrument

A simple way is to ask the patient whether there is somebody available to help in case of emergency or sickness [72].

## Conclusions

A selection of brief screening tests may help identify problems that are frequently overlooked in older patients. Results should trigger further assessments and management according to the patient’s beliefs, preferences, and expectations. Although no formal data exists to inform on the best timeline to repeat this basic evaluation, yearly assessments have been shown to have a good diagnostic yield. A formal evaluation of this two-step approach is still needed, but we strongly believe that such an approach is likely to yield some, if not all, of the benefits of community-based CGA programs, provided identified problems are properly addressed after the initial assessment.
